# Mitochondrial DNA copy number is associated with Crohn’s disease: a comprehensive Mendelian randomization analysis

**DOI:** 10.1038/s41598-023-48175-5

**Published:** 2023-11-29

**Authors:** Xianlei Cai, Xueying Li, Chao Liang, Miaozun Zhang, Yuan Xu, Zhebin Dong, Yihui Weng, Weiming Yu

**Affiliations:** 1 Department of Gastrointestinal Surgery, The Lihuili Affiliated Hospital, Ningbo University (Ningbo Medical Center Lihuili Hospital), Ningbo, 315000 Zhejiang China; 2 Department of Gastroenterology, The First Affiliated Hospital, Ningbo University, Ningbo, 315000 Zhejiang China

**Keywords:** Computational biology and bioinformatics, Genetics, Biomarkers, Gastroenterology, Risk factors

## Abstract

Mitochondrial DNA plays a critical role in the pathophysiological process of inflammation. However, the relationship between mitochondrial DNA copy number (mtDNA-CN) and inflammatory bowel diseases (IBD) remains poorly understood. We conducted a comprehensive Mendelian randomization (MR) using three instrumental variables (IVs) to explore the causal associations between mtDNA-CN and IBD, including Crohn's disease (CD), ulcerative colitis (UC). MR-Egger regression, weighted median, inverse-variance weighted (IVW), and weighted mode methods were used to evaluate the potential causal associations. The robustness of the IVW estimates was determined using the leave-one-out sensitivity test. A meta-analysis was conducted to pool the results from the three sets of IVs. Upon analysis, the findings of the current study revealed that genetically predicted mtDNA-CN was not associated with IBD (CD + UC) and UC. The results of MR analyses between mtDNA-CN and CD risk were inconsistent by using three sets of IVs. After a meta-analysis, we found that genetically predicted mtDNA-CN was associated with CD risk (odds ratio = 2.09; 95% confidence interval: 1.37–3.18). This finding was also confirmed by multivariable MR analyses and remained robust when tested with the leave-one-out sensitivity test. In conclusion, genetically predicted mtDNA-CN was found to be associated with CD risk. Therefore, mtDNA levels in the blood could potentially be used as a marker for CD risk assessment. Further studies are needed to elucidate the underlying mechanisms and validate the results of this study.

## Introduction

Inflammatory bowel diseases (IBDs) represent a chronic inflammatory disorder that affects the gastrointestinal tract, causing inflammation of the colon, rectum, and small intestine^1^. The two primary types of IBDs are Crohn's disease (CD) and ulcerative colitis (UC). CD can affect any part of the digestive system, causing patchy and transmural inflammation, which affect multiple layers of the intestinal wall, resulting in various symptoms, including fatigue, weight loss, diarrhea, abdominal pain, and others^2^. Conversely, UC is limited to the colon and rectum and causes continuous superficial inflammation, affecting only the innermost layer of the intestinal wall^3^. Although the specific cause of IBDs is unknown to date, it probably originates because of an abnormal immune response in people with a genetic predisposition^4^. In addition, environmental factors such as diet, smoking, and infections may contribute to the progression of the disease^5^. Sustained efforts have been catered by researchers to identify new therapies and better understand the underlying mechanisms of the disease to improve outcomes for individuals living with IBDs.

Mitochondria (MT), an essential cellular organelle in nearly every human cell, is primarily involved in producing energy through oxidative processes, signaling the apoptotic process, maintaining homeostasis, and synthesizing various metabolites^6,7^. Mitochondria contain their own genome, known as mitochondrial DNA (mtDNA), which is circular, intron-free, double-stranded, haploid, and approximately 16.6 kb^8^. The mitochondrial DNA copy number (mtDNA-CN) refers to the number of mtDNA copies per cell and it can be conveniently determined from peripheral blood^9^. In addition, mtDNA-CN is an easily attainable biomarker for mitochondrial function and health. Observational studies have indicated that those with a variation in mtDNA-CN are associated with age-related complex illnesses, including coronary artery disease^10^, stroke^11^, chronic kidney disease^12^, and hypertension^13^. In addition to aging and age-related disorders, the mtDNA level was found to be associated with cancer for both diagnostic and prognostic purposes^14^. However, to date, the relationship between mtDNA-CN and IBD remains poorly understood.

Genome-wide association studies (GWAS) have emerged as a valuable tool for analyzing vast genetic data from numerous individuals to identify genetic variants that may be associated with specific conditions or diseases. Mendelian randomization (MR) is a reliable epidemiological technique for verifying causal relationships between different exposures and outcomes. MR relies on the assumption that genetic variations are randomly distributed during conception and these variations can be used as instrumental variables (IVs) to accurately estimate the causal effects of exposures on outcomes, thereby preventing the possibilities of confounding or reverse causation. In recent years, the integration of MR and GWAS has been considerably popular, primarily owing to its superior performance compared to that of traditional epidemiological methods.

This study aimed to determine the causal relationship between mtDNA and IBDs using two-sample MR and multivariable MR (MVMR) analyses. The valuable findings regarding the relationship between mtDNA and IBDs will contribute to the development of strategies for IBD prevention and treatment.

## Methods

### MR design

**Figure 1:**
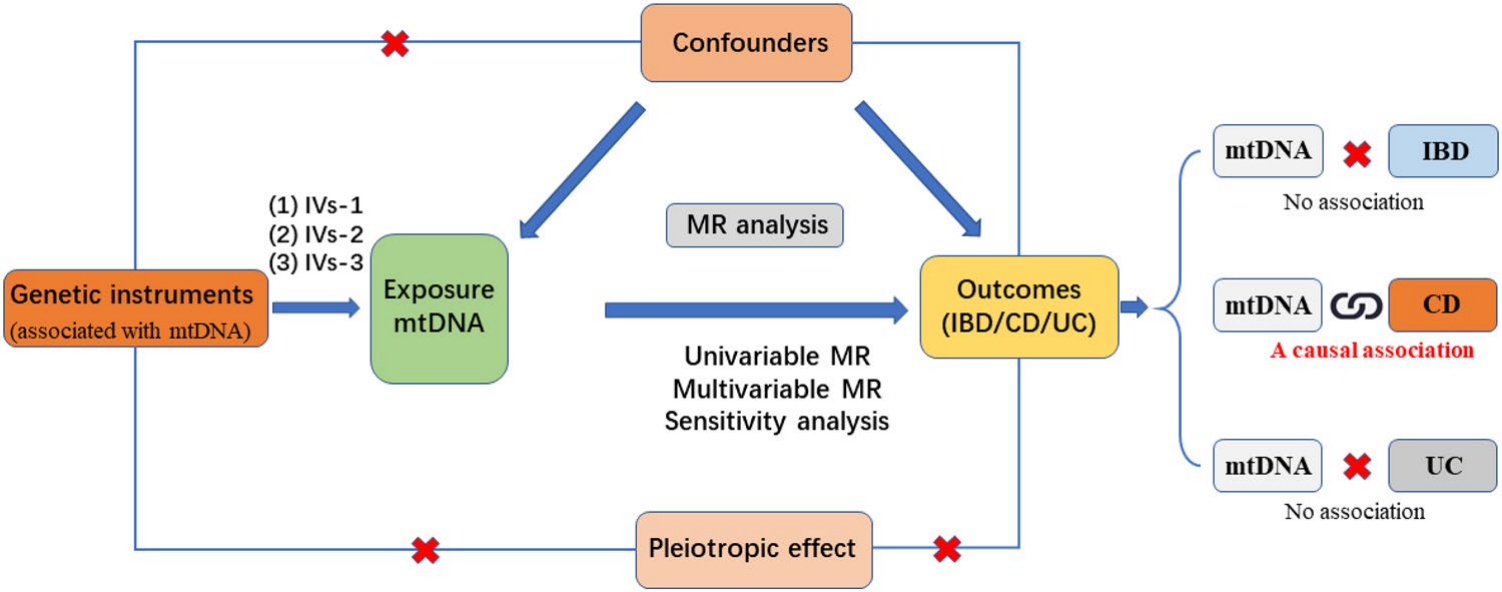
Flowchart of the data collection, processing, and analysis procedures of this study.

The STROBE-ME guidelines^15,16^ were followed during the current study. MR was designed based on three assumptions: (1) Relevance assumption: genetic variants are significantly associated with the mtDNA-CN; (2) Independence assumption: genetic variants are not associated with other factors that may confound IBD; and (3) Exclusion restriction assumption: genetic variants only influence IBDs by influencing the mtDNA-CN^17^ (Fig. 1).

### Selection of genetic variants

IVs were used to determine the potential associations between mtDNA-CN and IBDs. To obtain GWAS summary data, information on the IVs was collected from three sources: (1) IVs-1 were extracted from the study of Chong et al.^9^. They developed a novel method named ‘automatic mitochondrial copy (AutoMitoC)’ and applied it to 395,781 UK Biobank (UKB) study participants^18^ to identify novel common and rare genetic determinants for mtDNA-CN; (2) IVs-2 were extracted from the study of Hägg et al.^19^. They estimated mtDNA abundance from the weighted intensities of probes mapping to the MT genome in 295,150 participants from the UKB; and (3) IVs-3 were extracted from the study of Longchamps et al.^7^. They performed a GWAS in 465,809 White individuals from the Cohorts for Heart and Aging Research in the Genomic Epidemiology consortium and UKB. UKB is a large-scale, long-term project aimed at studying the genetic and environmental factors that affect human health. UKB recruits approximately 500,000 individuals from across the United Kingdom to provide detailed health, lifestyle, and genetic information^20^, which is stored and made available to researchers worldwide for health research purposes. The database is a valuable resource for medical research, with the aim of improving diagnosis, treatment, and prevention of diseases.

To ensure the quality of the instrumental single nucleotide polymorphisms (SNPs) used for mtDNA-CN, a set of quality control measures was implemented. First, the SNPs significantly associated with mtDNA-CN at the traditional threshold (*P* < 5 × 10^–8^) were extracted. Second, only the SNPs with a low likelihood of linkage disequilibrium estimates (r^2^ < 0.001) and a long physical distance (window size = 10,000 kb) from European samples in the 1000 Genomes Project were retained. Third, the SNPs with a minor (< 0.01) allele frequency (MAF) were removed. In addition, the proportion of variance explained by the SNPs (PVE) was evaluated using the equation: *PVE* = 2 × *MAF* ×  (1* − MAF*) × *beta*^2^^21^, and the *F*-statistic was calculated to determine the strength of SNP using the equation: *F* = *PVE* ×  (*N − *2)/ (*1 − PVE*)^21^. In the cases where MAF data were not provided in the original studies, an alternative formula was employed: *F* = *beta*^2^*/se*^2^^22^. The statistical power of *F* > 10 indicated a strong association^17^.

Any candidate genetic tools that did not pass the MR pleiotropy residual sum and outlier (MR-PRESSO) test with a significance level of less than 0.05 were removed^23^. This rigorous screening process enhances the reliability of the results of this study. An overview of the three genetic tools used in the current study is provided in Supplementary Table 1. Ethical approval was obtained in the original studies.

### GWAS summary statistics of IBDs

FinnGen is a Finnish Genome Project dataset that includes genetic and phenotypic information on Finnish individuals. The dataset was created by combining multiple Finnish population-based cohorts, including participants from all over Finland, with high-quality genotyping data^24^. FinnGen is a unique resource for genetic research and has been used for various studies on common diseases and traits. The FinnGen dataset is also characterized by its high-quality data curation and stringent quality control measures, ensuring its accuracy and reproducibility for research purposes. This dataset has been made available to researchers worldwide for the purpose of enhancing understanding of the genetics underlying health and disease. To ensure comparability in patient ancestry, the causal association of mtDNA-CN with IBDs was explored using summary statistics derived from the FinnGen dataset through the Integrative Epidemiology Unit (IEU) Open GWAS project^25^. The FinnGen Biobank GWAS, performed by the FinnGen team (https://finngen.gitbook.io/documentation/v/r5/data-download), involved 3,753 IBD cases and 210,300 genetically matched controls, 848 CD cases and 217,852 genetically matched controls, and 2,578 UC cases and 215,806 genetically matched controls. In the IEU open GWAS platform, the GWAS IDs corresponding to IBD, CD, and UC were “finn-b-K11_IBD_STRICT,” “finn-b-K11_CD_STRICT2,” and “finn-b-K11_UC_STRICT2,” respectively. Although both the UKB and FinnGen datasets contain approximately 1,000,000 SNPs, exhibiting a high level of consistency, there are still inconsistencies in SNPs between the exposure and outcome datasets. During the extraction of SNPs associated with the exposure from the outcome, any SNPs without relevant information in the outcome were eliminated^26^.

The participant overlap in MR analysis can cause inflated type I errors^27^. Because the exposure factors were mainly derived from UKB, the GWAS of IBD cases based on the UKB database were excluded to avoid bias due to sample overlap.

### Statistical analysis

The step-by-step approach devised for conducting MR is presented as a flowchart in Fig. 1. Initially, a matching index was used to harmonize the GWAS data of mtDNA-CN and IBDs with the selected IVs. Subsequently, any pleiotropic outliers among the selected IVs were eliminated using the MR-PRESSO method before MR analysis. Finally, MR-Egger regression was applied to assess horizontal pleiotropy and any evidence of its absence, indicated by a P-value larger than 0.05^28^.

After eliminating pleiotropy, Cochran's Q test was performed to detect heterogeneity among SNPs. Various MR methods were employed to ensure consistency in the directions [i.e., MR-Egger regression, weighted median, inverse variance weighted (IVW; fixed-effects or random-effects models), and weighted mode (WM)]. The obtained results were visualized using scatter plots. To measure the odds ratios (ORs) and corresponding 95% confidence intervals (CIs) for IBDs, the ORs and corresponding 95% CIs of IBDs were calculated per one-standard deviation (SD) increase^23^. Finally, a leave-one-out sensitivity test was performed to evaluate the robustness of the IVW estimates. In the sensitivity analysis, we systematically omitted one SNP in turn and recalculated the data. If the statistical significance remained consistent across all the results, it indicated that the finding was robust. Conversely, if the statistical significance of the outcomes changed after the omission of a specific SNP, it suggested that this SNP has a significant impact on the outcomes.

To facilitate the integration of the IVs from the three sets, a meta-analysis was conducted to consolidate the findings. Heterogeneity was evaluated by Q-test and *I*^2^ test. For the results that demonstrated notable heterogeneity (*p* ≤ 0.05 and *I*^2^ > 50%), pooled results were calculated using DerSimonian and Laird method^29^. Otherwise, the Mantel–Haenszel method was used^30^.

MVMR is an important auxiliary approach that utilizes genetic variants to assess the impact of different exposures on a specific outcome^31^. It is well established that smoking is the most common risk factor for IBD^5^. In order to ensure a more conservative conclusion, MVMR was applied to account for the genetic association between the IVs used in the study and smoking. This adjustment was made in cases where the two-sample MR analysis yielded positive results. In the IEU open GWAS platform, the GWAS ID corresponding to smoking was “ukb-d-20116_0”.

*P* < 0.05 was applied as a significant causal association between mtDNA-CN and IBDs. MR analyses were performed using the TwoSampleMR function of the R package (version 4.0.3). Furthermore, meta-analysis was performed using STATA (version 12.0, StataCorp LP).

### Ethics approval and consent to participate

Our analyses were based on publicly available data that have been approved by relevant review boards and no additional ethical approval and consent to participate is required.

## Results

### Baseline participant characteristics

Three sets of IVs were evaluated to investigate the causal relationships between mtDNA-CN and IBD, CD, and UC. The number of SNPs of “IVs-1”, “IVs-2” and “IVs-3” was 55, 47, and 78, respectively after quality control. The explained variances were relatively small, and upon analysis using the *F* statistics for each chosen SNP, all variances exceeded 10, indicating a lack of potential weak instrument bias (Supplementary Table 1). The results of the MR-PRESSO test, horizontal pleiotropy test, heterogeneity test, and three MR methods are presented in Table 1.

**Table 1:** MR analyses of the causal effect of mtDNA-CN on IBDs.

Trait	SNP	MR-Egger		Weighted median		IVW/WM		MR-PRESSO	Pleiotropy	Q	Heterogeneity
		OR (95% CI)	*p*	OR (95% CI)	*p*	OR (95% CI)	*p*	Global test *P*	*P*		*P*
Instrumental variables 1	55										
IBD		0.72 (0.31–1.65)	0.4366	0.87 (0.49–1.54)	0.6308	1.10 (0.74–1.64)	0.6360	0.065	0.256	55.47	0.080
CD		2.00 (0.37–10.83)	0.4242	1.55 (0.50–4.77)	0.4415	2.85 (1.31–6.22)	0.0086	0.076	0.646	59.24	0.076
UC		0.50 (0.20–1.25)	0.1466	0.70 (0.67–1.34)	0.1943	0.74 (0.48–1.16)	0.1943	0.247	0.336	45.97	0.274
Instrumental variables 2	47										
IBD		1.59 (0.46–5.49)	0.4651	0.72 (0.42–1.25)	0.2469	1.15 (0.77–1.71)	0.4946	0.164	0.587	47.00	0.178
CD		10.29 (0.84–125.51)	0.0753	1.47 (0.46–4.67)	0.5159	1.61 (0.70–3.69)	0.5159	0.102	0.132	51.38	0.107
UC		0.65 (0.17–2.52)	0.5384	0.77 (0.41–1.45)	0.4150	0.81 (0.52–1.28)	0.3757	0.755	0.732	29.81	0.757
Instrumental variables 3	78										
IBD		0.63 (0.33–1.22)	0.1735	0.89 (0.55–1.46)	0.6537	0.88 (0.63–1.23)	0.4673	0.148	0.247	66.32	0.163
CD		2.18 (0.60–7.87)	0.2397	1.16 (0.44–3.10)	0.7638	1.97 (1.04–3.70)	0.0486	0.205	0.861	68.25	0.217
UC		0.55 (0.26–1.16)	0.1238	0.72 (0.39–1.33)	0.2965	0.73 (0.50–1.06)	0.0989	0.255	0.404	60.97	0.302

### Association between mtDNA-CN and IBD risk

**Figure 2:**
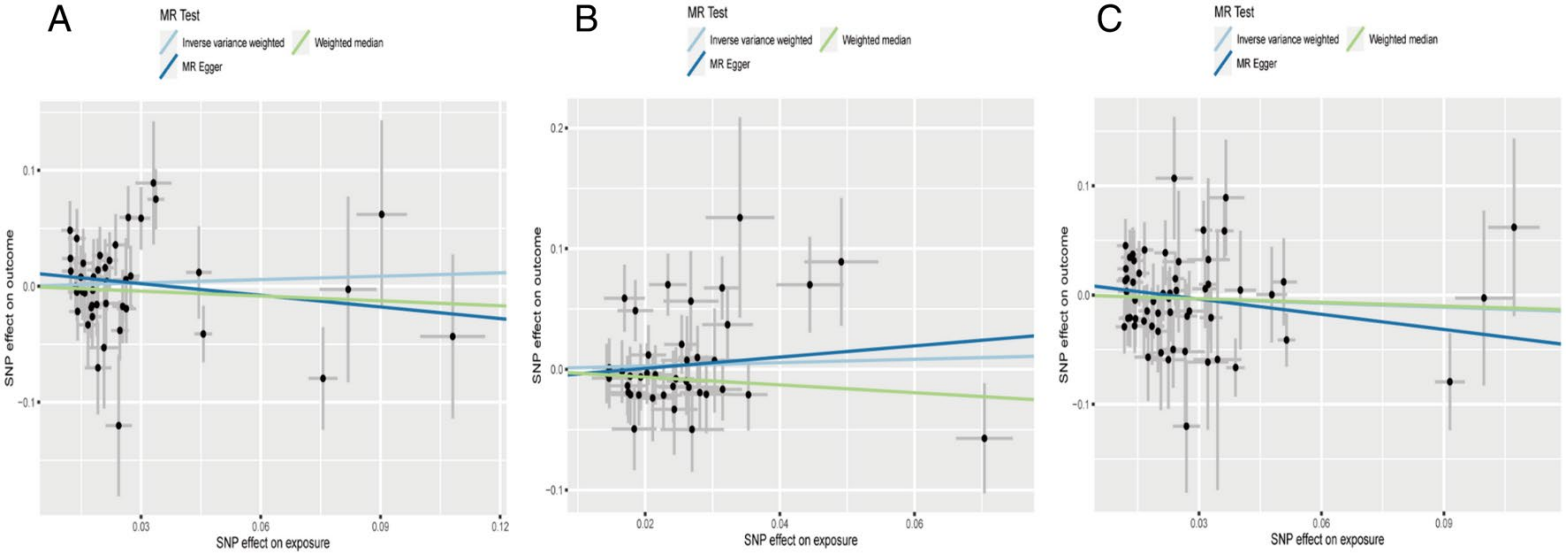
The scatter plots for MR analyses of IBD: (**A**) IVs-1; (**B**) IVs-2; (**C**) IVs-3.

**Figure 3:**
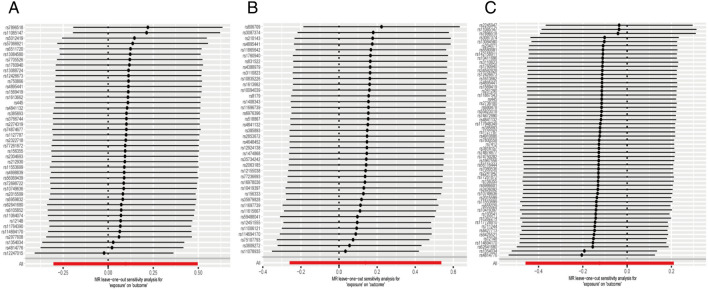
The sensitive analyses for MR analyses of IBD: (**A**) IVs-1; (**B**) IVs-2; (**C**) IVs-3.

Regarding “IVs-1”, MR analysis revealed the absence of any causal association between mtDNA-CN and IBD risk (OR = 1.10; 95% CI: 0.74–1.64; *P* = 0.636). No pleiotropy and heterogeneity were observed (*P*_pleiotropy_ = 0.256; *P*_heterogeneity_ = 0.080) after the MR-PRESSO test. The scatter plot for MR analysis is presented in Fig. 2A, and the funnel plot is presented in Supplementary Fig. 1A. Further, the leave-one-out sensitivity test indicated robust results (Fig. 3A).

Regarding “IVs-2”, MR analysis revealed results similar to that with “IVs-1” (OR = 1.15; 95% CI: 0.77–1.71; *P* = 0.495). No pleiotropy and heterogeneity were observed after the MR-PRESSO test (*P*_pleiotropy_ = 0.587; *P*_heterogeneity_ = 0.178). The scatter plot for MR analysis is presented in Fig. 2B, and the funnel plot is presented in Supplementary Fig. 1B. The leave-one-out sensitivity test indicated robust results (Fig. 3B).

Regarding “IVs-3”, the absence of any association between mtDNA-CN and IBD risk (OR = 0.88; 95% CI: 0.63–1.23; *P* = 0.467) was confirmed. No pleiotropy and heterogeneity were observed after the MR-PRESSO test (*P*_pleiotropy_ = 0.247; *P*_heterogeneity_ = 0.163). The scatter plot for MR analysis is presented in Fig. 2C, and the funnel plot is presented in Supplementary Fig. 1C. The leave-one-out sensitivity test indicated robust results (Fig. 3C).

### Association between mtDNA-CN and CD risk

**Figure 4:**
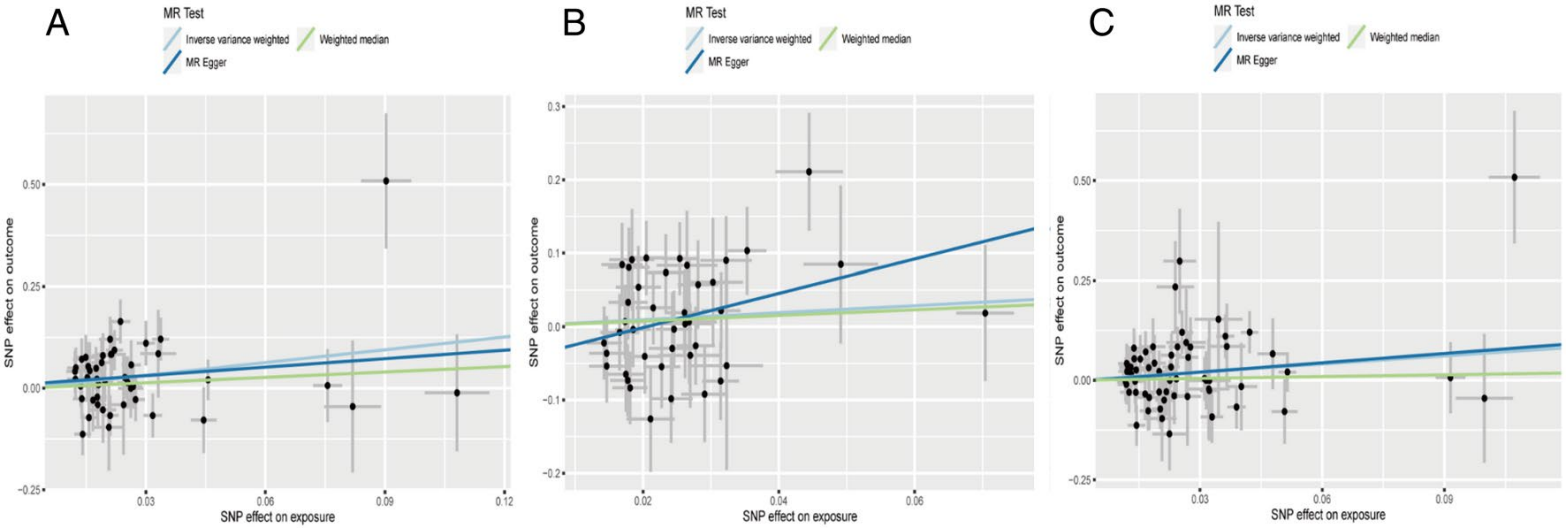
The scatter plots for MR analyses of CD: (**A**) IVs-1; (**B**) IVs-2; (**C**) IVs-3.

**Figure 5:**
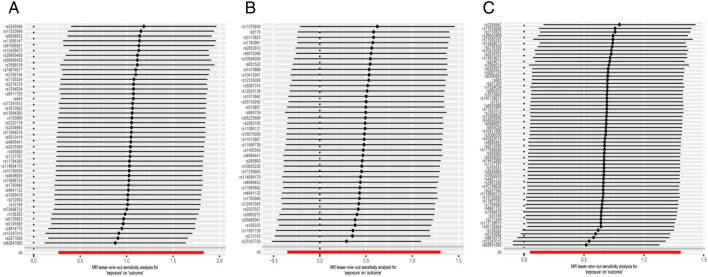
The sensitive analyses for MR analyses of CD: (**A**) IVs-1; (**B**) IVs-2; (**C**) IVs-3.

Regarding “IVs-1”, MR analysis based on the IVW method revealed a causal effect of mtDNA-CN on CD risk per 1-SD increase in mtDNA-CN (OR = 2.85; 95% CI: 1.31–6.22; *P* = 0.009). No pleiotropy and heterogeneity were observed (*P*_pleiotropy_ = 0.646; *P*_heterogeneity_ = 0.076 after the MR-PRESSO test. The scatter plot for MR analysis and funnel plot are presented in Fig. 4A and Supplementary Fig. 2A, respectively. Further, the leave-one-out sensitivity test indicated robust results (Fig. 5A).

Regarding “IVs-2”, no evidence for a causal association between mtDNA-CN and CD was found (OR = 1.61; 95% CI: 0.70–3.69; *P* = 0.516). In addition, no pleiotropy and heterogeneity were observed after the MR-PRESSO test (*P*_pleiotropy_ = 0.132; *P*_heterogeneity_ = 0.107). The scatter plot for MR analysis is presented in Fig. 4B, and the funnel plot is presented in Supplementary Fig. 2B. The leave-one-out sensitivity test indicated robust results (Fig. 5B).

Regarding “IVs-3”, the available evidence suggested a causal association between mtDNA-CN and CD (OR = 1.97; 95% CI: 1.04–3.70; *P* = 0.049). No pleiotropy and heterogeneity were observed after the MR-PRESSO test (*P*_pleiotropy_ = 0.861; *P*_heterogeneity_ = 0.217). The scatter plot for MR analysis and funnel plot are presented in Fig. 4C and Supplementary Fig. 2C, respectively. The leave-one-out sensitivity test indicated that the positive result was influenced by rs156355, rs5745582, rs4814776, rs12247015, and rs62641680 (Fig. 5C).

As the univariate MR analysis yielded positive results for “IVs-1” and “IVs-3”, we conducted MVMR analysis to account for the effect of smoking on mtDNA-CN, the most common risk factor for CD. The MVMR results also indicated significant associations between mtDNA-CN and CD (OR = 3.62; 95% CI: 1.61–8.18 for “IVs-1”; OR = 2.16; 95% CI: 1.14–4.11 for “IVs-3”). These findings provide further support for the robustness of the current study.

### Association between mtDNA-CN and UC risk

**Figure 6:**
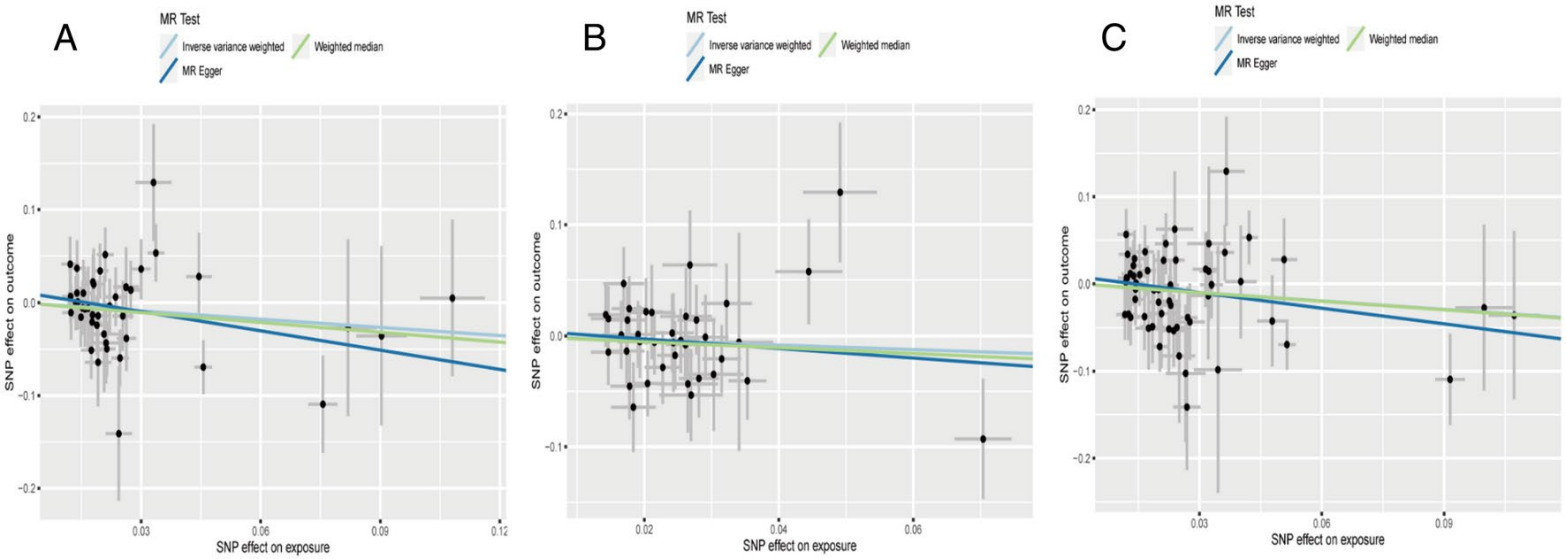
The scatter plots for MR analyses of UC: (**A**) IVs-1; (**B**) IVs-2; (**C**) IVs-3.

**Figure 7:**
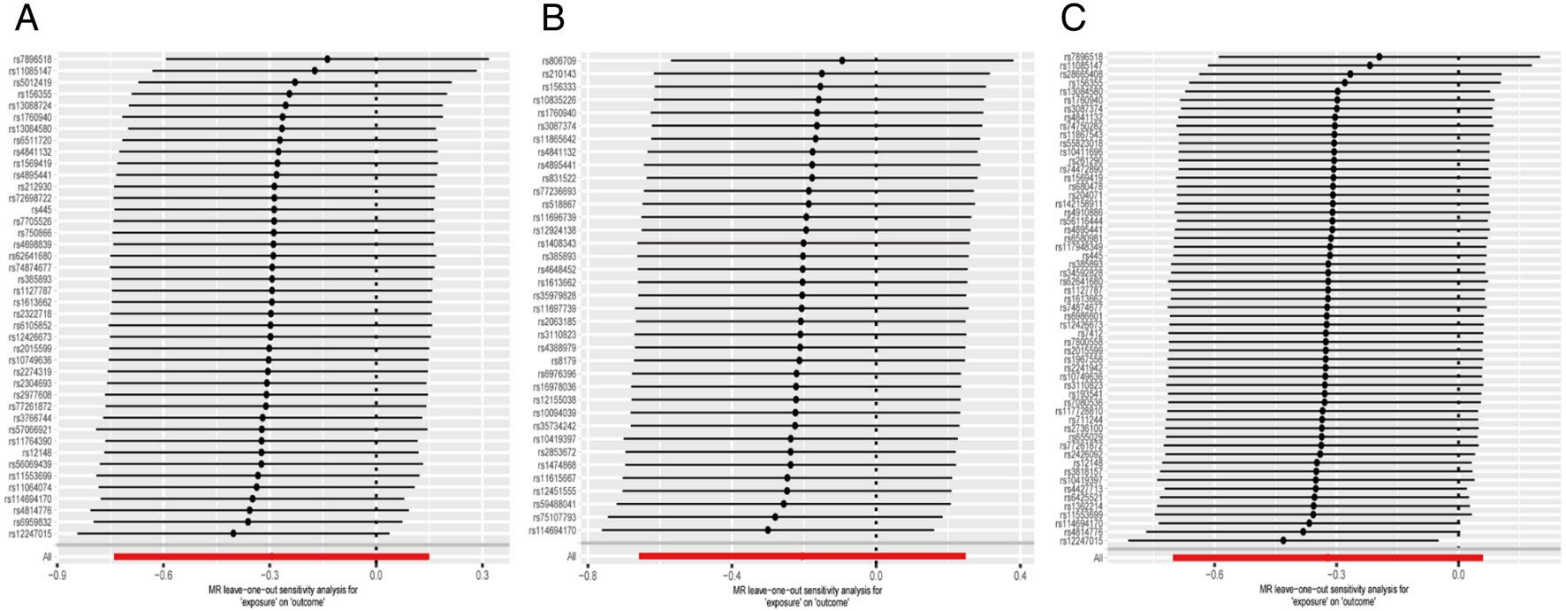
The sensitive analyses for MR analyses of UC: (**A**) IVs-1; (**B**) IVs-2; (**C**) IVs-3.

Regarding “IVs-1”, MR analysis revealed the absence of any causal association between mtDNA-CN and UC risk (OR = 0.74; 95% CI: 0.48–1.16; *P* = 0.194). In addition, no pleiotropy and heterogeneity were observed (*P*_pleiotropy_ = 0.336; *P*_heterogeneity_ = 0.274) after the MR-PRESSO test. The scatter plot for MR analysis and funnel plot are presented in Fig. 6A and Supplementary Fig. 3A, respectively. Moreover, the leave-one-out sensitivity test indicated robust results (Fig. 7A).

Regarding “IVs-2”, MR analysis yielded results similar to that of “IVs-1” (OR = 0.81; 95% CI: 0.52–1.28; *P* = 0.376). Moreover, no pleiotropy and heterogeneity were observed after the MR-PRESSO test (*P*_pleiotropy_ = 0.732; *P*_heterogeneity_ = 0.757). The scatter plot for MR analysis is presented in Fig. 6B, and the funnel plot is presented in Supplementary Fig. 3B. The leave-one-out sensitivity test indicated robust results (Fig. 7B).

Regarding “IVs-3”, the absence of association between mtDNA-CN and UC risk (OR = 0.73; 95% CI: 0.50–1.06; *P* = 0.099) was confirmed. No pleiotropy and heterogeneity were observed after the MR-PRESSO test (*P*_pleiotropy_ = 0.404; *P*_heterogeneity_ = 0.302). The scatter plot for MR analysis and funnel plot are presented in Fig. 6C and Supplementary Fig. 3C. The leave-one-out sensitivity test indicated robust results (Fig. 7C).

### Meta-analysis of the MR results across the three IV sets

**Figure 8:**
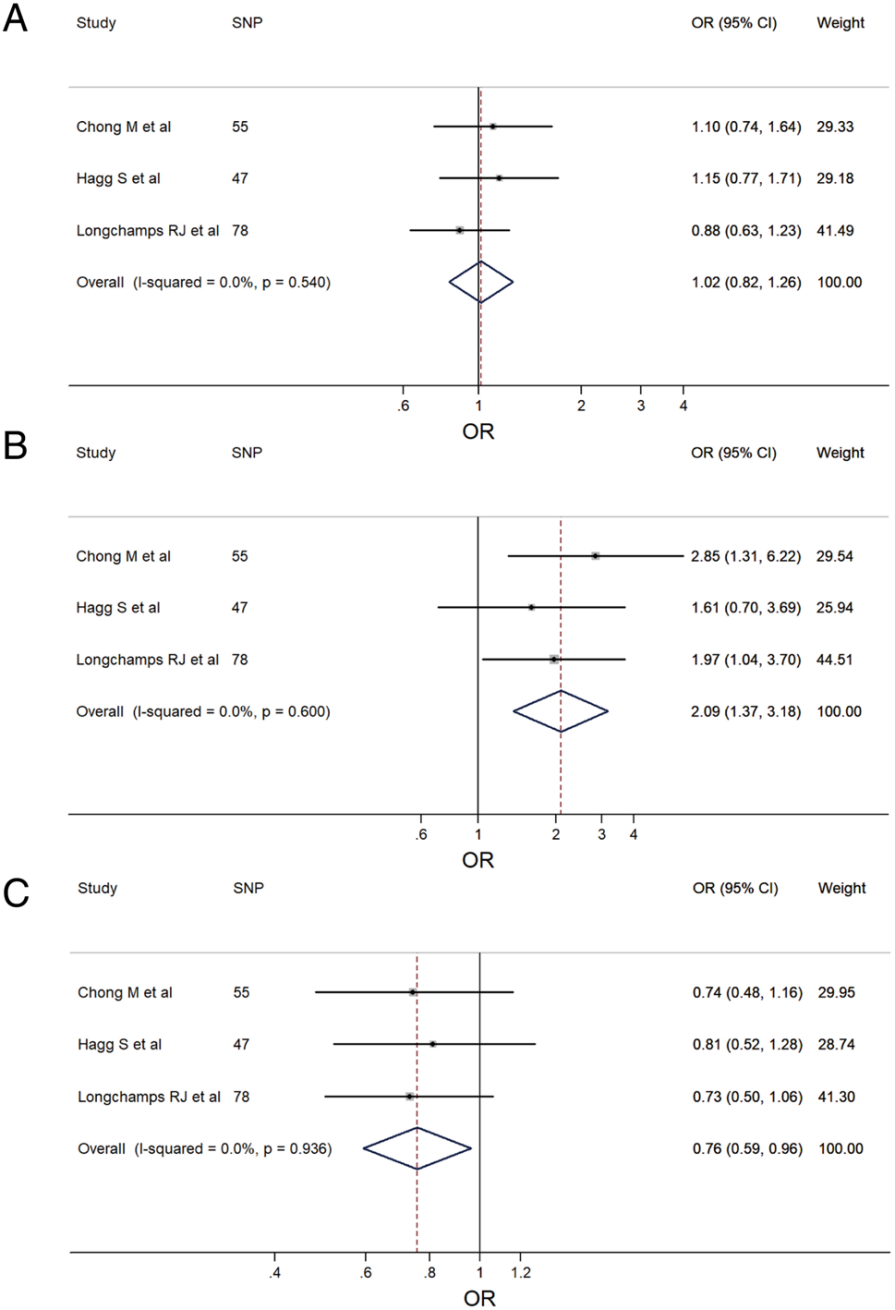
The forest plots of meta-analysis: (**A**) IBD; (**B**) CD; (**C**) UC.

Owing to inconsistent results obtained from the MR analyses conducted across the three different IV sets, a meta-analysis was performed. With regards to IBD, the meta-analysis results confirmed that there was no association between mtDNA-CN level and IBD risk (pooled OR = 1.02; 95% CI: 0.82–1.26; Fig. 8A) without any heterogeneity observed (*P* = 0.540; I^2^ = 0.0%). In the case of CD, the meta-analysis results indicated that an increase in mtDNA-CN among participants led to a higher risk of CD (pooled OR = 2.09; 95% CI: 1.37–3.18; Fig. 8B) without any heterogeneity observed (*P* = 0.600; I^2^ = 0.0%). For UC, the meta-analysis results showed that an increase in mtDNA-CN among participants decreased the risk of UC (pooled OR = 0.76; 95% CI: 0.59–0.96; Fig. 8C) without any heterogeneity observed (*P* = 0.936; I^2^ = 0.0%). However, despite the statistical significance of the results, all three estimates included in the meta-analysis were negative. Additionally, sensitivity analyses revealed that regardless of which set of IVs was omitted, the result remained negative (Supplementary Fig. 4). Therefore, we concluded that this result should be interpreted cautiously.

## Discussion

In this study, the causal effect of mtDNA-CN on the risk of IBDs was evaluated through univariable and multivariable MR analyses. To the best of our knowledge, the MR analysis in this study is the first one that focuses on the topic. The current study showed that genetically predicted mtDNA-CN is an important indicator strongly associated with CD, whereas it has no potential association with UC. Both the heterogeneity tests of MR analysis and meta-analysis indicated that all the estimates were homogeneous, which reduced the bias and improved the reliability of the results. These findings are essential for a better understanding of mitochondrial function in patients with CD.

We identified some overlap among the three sets of IVs. Specifically, seven SNPs (rs4841132, rs385893, rs114694170, rs1760940, rs4895441, rs1613662, rs342293) were present in all three sets of IVs (Supplementary Fig. 5), indicating that these seven SNPs may play a critical role and warrant further investigation into their molecular mechanisms. Due to the different calculation methods used to derive the three IV sets, the overlapping SNPs exhibited varying effect sizes and standard errors. According to the descriptions in the three original articles, we found the three sets of IVs have the same directions. In addition, we applied the harmonization function to assess the congruence of SNP allelic orientation between the exposure and outcome. Moreover, we conducted a meta-analysis to merge the results and obtain more reliable findings, following the method described by Xie et al.^22^.

Boyapati et al.^32^ reported an observational study with 30 patients with CD and 40 non-IBD controls, and they found that plasma mtDNA levels were increased in CD compared with that in the non-IBD controls. The findings of this study align with the results of the current study. However, only a limited number of studies exist that assessed the relationship between mtDNA and IBDs. Further studies are needed to validate or update the results of the current.

IBD is a serious threat to the global population, which increases the social burden. The chronic inflammation and damage caused by IBD lead to a range of complications, including strictures, fistulas, and abscesses, as well as an increased risk of colorectal cancer^33^. In addition, IBD can significantly impact the quality of life, with symptoms such as abdominal pain, diarrhea, and fatigue leading to social isolation, depression, and anxiety^34,35^. In severe cases, IBD can be life-threatening, particularly if complications such as bowel perforation or sepsis occur. Moreover, IBD is associated with a higher risk of developing other serious conditions^36^, including osteoporosis^37^, primary sclerosing cholangitis^38^, and cardiovascular disease^39^. Owing to its chronic nature, IBD requires continuous management and monitoring by healthcare providers, and patients often require long-term medication and/or surgery to manage symptoms and prevent complications. Thus, IBD poses significant risks to both physical and mental health, highlighting the need for advanced treatment options to improve outcomes for affected individuals.

It is to be noted that the etiology of IBD remains elusive to date. The epidemiological evolution of IBD suggests that the westernization of lifestyle and industrialization is linked to the emergence of IBD in developing nations over the past decades^40^. Urbanization causes changes in diet, antibiotic use, microbial exposure, and pollution, which are all possible environmental risk factors for IBD^41,42^. The role of the gut microbiota in the development and propagation of inflammation in IBD highlights the significance of environmental influences in the disease^43^. It is crucial to comprehend the risk factors in inflammatory bowel disease (IBD) as it may assist in identifying ways to decrease the likelihood of contracting the disease or to alleviate its severity. Moreover, the quest for pathogenic risk factors is important, as several suboptimal outcomes and therapeutic needs in IBD remain to date. Further, a better understanding of the underlying mechanisms can be obtained by accurately defining the impact of innovative risk factors, thereby resulting in the identification of new therapeutic targets and treatment approaches.

Abnormal regulation of the immune system plays a critical role in the maintenance of intestinal homeostasis and the development of IBD^44^. The innate immune functionality in the intestine is primarily performed by neutrophils, monocytes, macrophages, dendritic cells, innate lymphoid cells, and natural killer cells, which provide rapid and nonspecific defense against pathogens and the excessive entry of intestinal microorganisms^45^. The dysregulation of this equilibrium is linked to intestinal inflammation and IBD^46^. Innate immune cells participate in host defense responses, inflammation, and tissue healing, which is achieved through the production of cytokines and chemokines, initiation of the complement pathway and phagocytosis, as well as presentation of antigens to activate adaptive immune responses^47^. They play critical roles in promoting or ameliorating the cellular and molecular mechanisms sustaining IBD.

mtDNA expression has been determined as an essential factor for the biogenesis of the oxidative phosphorylation system. To cope with the metabolic requirements, the adjustment of the mtDNA content in cells and tissues is necessary^14^. The regulation of mtDNA levels is a complex process that involves a delicate balance of replication and turnover. Mitochondrial diseases are a varied set of genetic disorders marked by a deficiency in the oxidative phosphorylation system. These disorders can affect diverse cell types and organs, causing various symptoms. Mitochondrial diseases are caused either by mutations in nuclear genes that result in reduced mtDNA expression, or by primary mtDNA mutations that impair the function or abundance of mtDNA^14^.

Collins et al. first reported the immunostimulatory potential of mtDNA^48^. Thereafter, numerous studies have corroborated the initial findings and provided further evidence that mtDNA could directly interact with pattern recognition receptors (PRRs) of the innate immune system, which subsequently results in an amplified pro-inflammatory response^49^. Inflammasomes are specialized protein complexes found within the cytoplasm of cells, that contain multiple subunits comprising receptor and sensor molecules. Substantial evidence indicated mtDNA as an endogenous inflammasome agonist^50^. Furthermore, recent evidence showed that both cytosolic as well as extracellular mtDNA activate PRRs, leading to the induction of type I interferons (IFNs) and subsequently enhancing the expression of interferon-stimulated genes. Boyapati, et al.^32^ also suggested mtDNA-Toll-like receptor 9 (TLR9) to be a therapeutic target in IBD. The current study revealed that high mtDNA-CN increased the risk of CD. Although the molecular mechanism by which mtDNA contributed to this increased risk was not well understood, it is hypothesized that the regulatory method of mtDNA/immune response/IBD could explain the current observations. However, any association between mtDNA and UC was not revealed in the current study. In addition, although both CD and UC are classified as IBDs, some differences exist in the pathogenesis and risk factors of the two.

To comprehend the causal effect of mtDNA variations in relation to IBDs, the precise and meticulous evaluation of mtDNA-CN is necessary. This is essential to remove any potential bias that may lead to the misinterpretation of outcomes. In the current study, three sets of IVs with different assessment methods were included to reduce the potential biases. Chong, et al^9^. developed a novel method (AutoMitoC) to estimate mtDNA-CD from genetic array data to identify common and rare genetic determinants. Longchamps et al.^7^ performed a GWAS to identify 133 SNPs with statistically significant effects associated with mtDNA-CN. Hägg et al.^19^ also identified 50 independent SNPs significantly associated with mtDNA abundance. However, this work did not account for platelets as a significant confounding factor in relation to blood mtDNA-CN levels, and this is a potential source of bias. We used a meta-analysis to pool the inconsistent results produced by these three sets of IVs to reduce the potential bias.

Despite providing some valuable insights, the current study also exhibits several limitations. First, only the GWAS data of mtDNA-CN for the European population was included. Although a high incidence of IBDs is common in Asia, the use of IVs across ancestry in MR analysis is inappropriate and it may introduce indeterminate bias. Therefore, relevant MR analysis in the Asian population was not performed. The large-scale GWAS data of mtDNA-CN for the Asian population needs to be reported in the future. Second, the sample size of IBDs in the FinnGen dataset is not adequately large, especially for CD. A larger GWAS database containing sufficient IBD cases is necessary to overcome case deficiency. Third, although three sets of IVs from three independent articles with different statistical approaches were included, the source data for these IVs came from UK Biobank. The new IVs of mtDNA-CN from other independent GWAS database is necessary to validate or update the results of the current study. Forth, owing to the lack of sufficient SNP data for mtDNA-CN, the bidirectional MR analysis was not performed.

## Conclusion

The comprehensive MR analysis in this study demonstrated that genetically predicted mtDNA-CN is associated with CD risk but not with UC risk. Moreover, the mtDNA levels in the blood have the potential to act as a marker for CD risk assessment. Therefore, the therapy targeting mtDNA may improve outcomes in CD patients.

### Supplementary Information

Supplementary Information.

## Data Availability

The datasets used and/or analyzed during the current study are presented in the manuscript. Summary statistics for GWAS are publicly available.
