# Supplementary material for: Mitochondrial DNA copy number is associated with Crohn’s disease: a comprehensive Mendelian randomization analysis
Source: Sci Rep. 2023 Nov 29;13:21016. doi: 10.1038/s41598-023-48175-5 (PMC10687096; doi:10.1038/s41598-023-48175-5)
Supplement: Supplementary file 1 — Supplementary Information. [file 41598_2023_48175_MOESM1_ESM.docx]

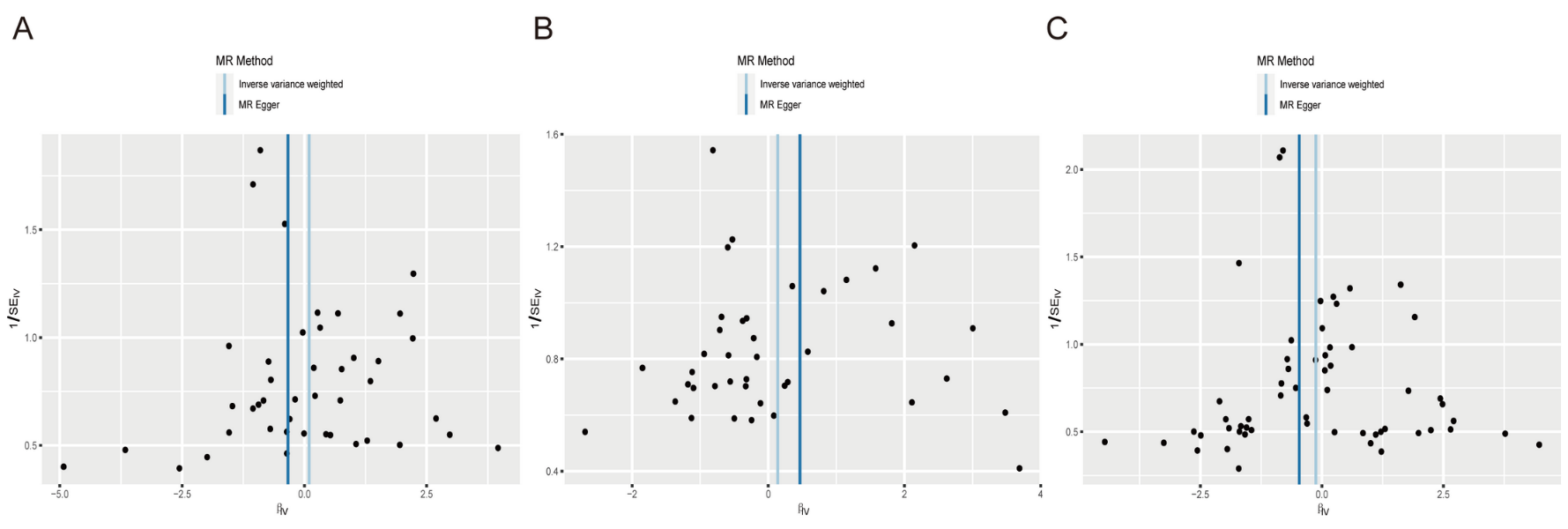


**Supplementary Figure 1. The funnel plots for MR analyses of IBD: (A) IVs-1; (B) IVs-2; (C) IVs-3.**


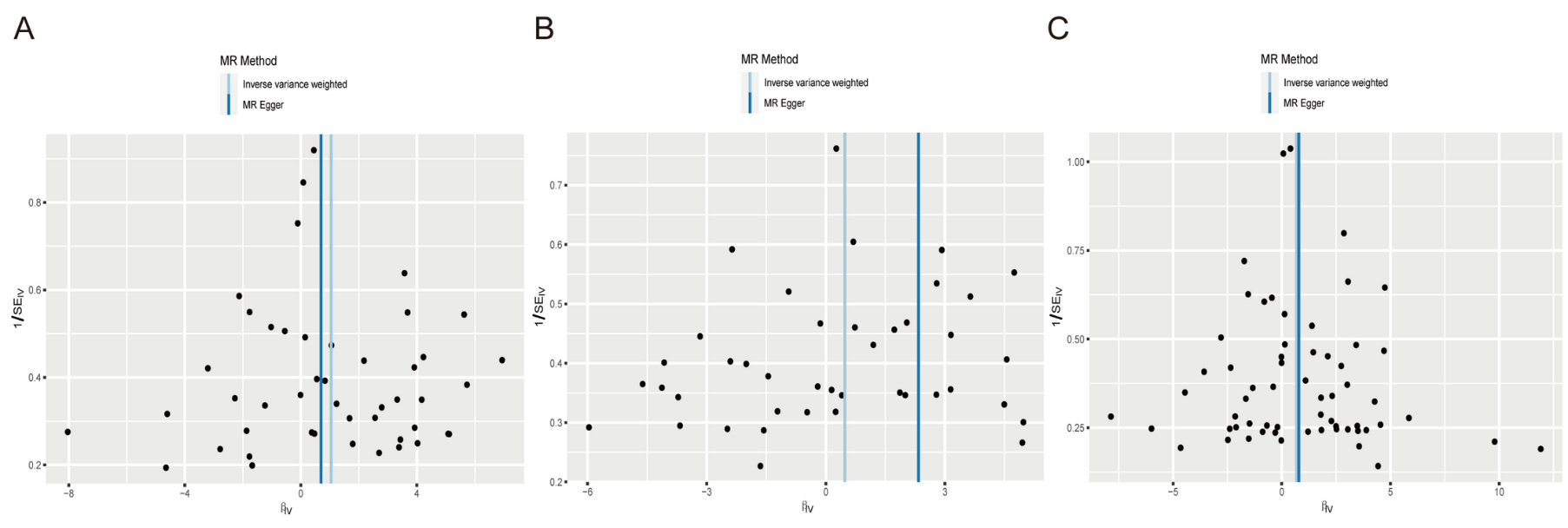


**Supplementary Figure 2. The funnel plots for MR analyses of CD: (A) IVs-1; (B) IVs-2; (C) IVs-3.**


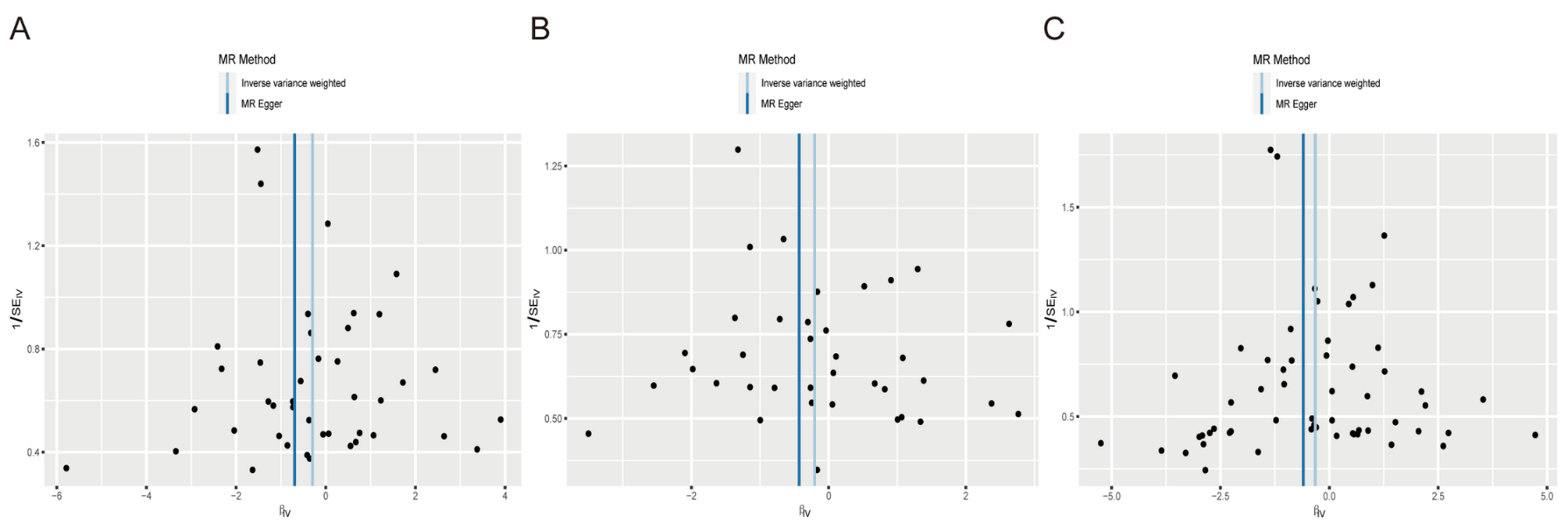


**Supplementary Figure 3. The funnel plots for MR analyses of UC: (A) IVs-1; (B) IVs-2; (C) IVs-3.**


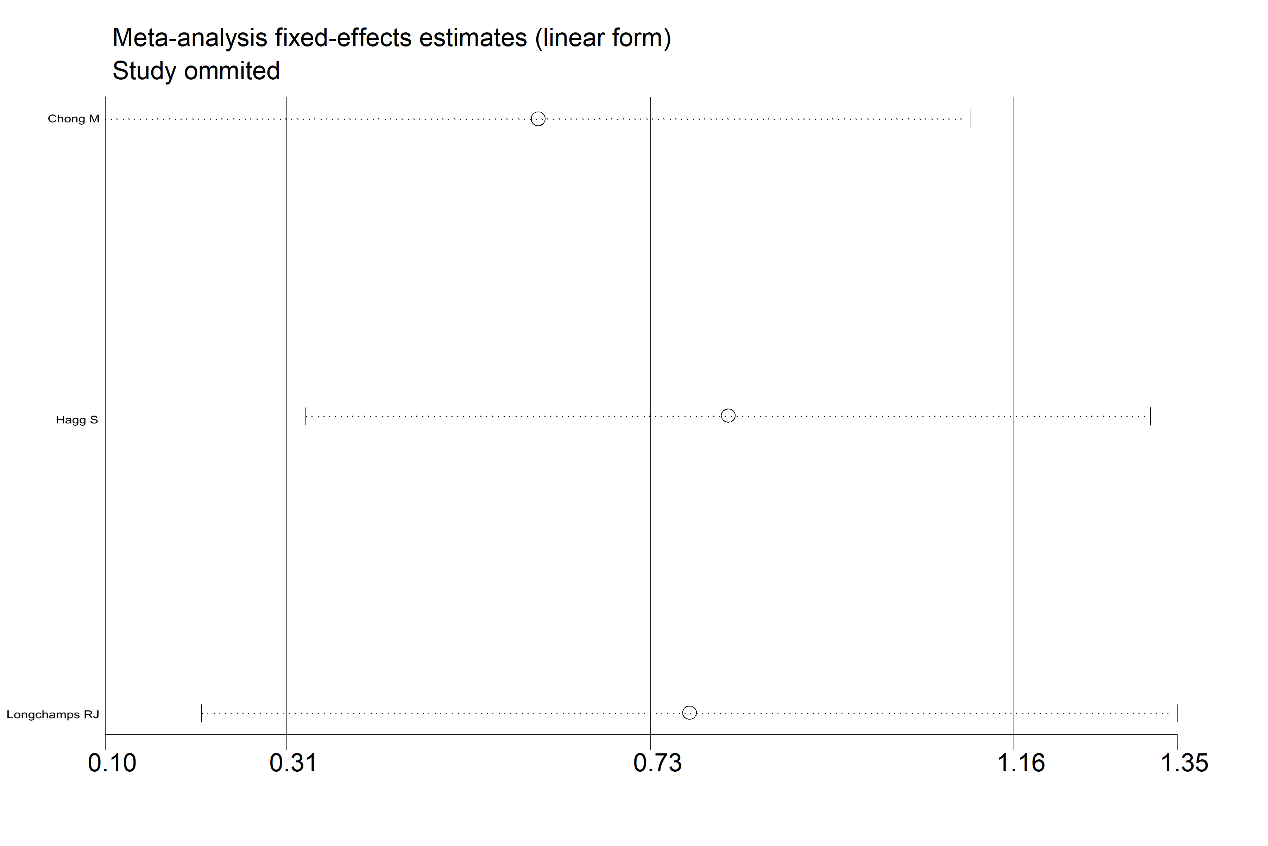


**Supplementary Figure 4. Sensitive analysis of Meta-analysis for the association between the mtDNA-CN and UC risk.**


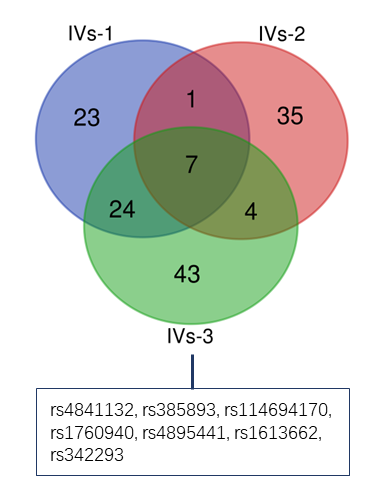


**Supplementary Figure 5. The Venn diagram of three IVs sets.**

**Supplementary Table 1. The genetic variants used as instrument variables**

| **Exposure** | **rsID** | **EA** | **OA** | **EAF** | **Beta** | **SE** | **Pval** | **Sample size** | **R2** | **F statistic** | **Study** |
| --- | --- | --- | --- | --- | --- | --- | --- | --- | --- | --- | --- |
| **Instrumental variables 1** | |  |  |  |  |  |  |  |  |  |  |
|  | rs2977608 | C | A | NA | 0.0236 | 0.0025 | 2.58E-21 | 395781 | NA | 89.11 | Chong M et al |
|  | rs1569419 | C | T | NA | 0.0189 | 0.0025 | 6.87E-14 | 395781 | NA | 57.15 | Chong M et al |
|  | rs3766744 | A | G | NA | -0.0179 | 0.0021 | 2.91E-17 | 395781 | NA | 72.66 | Chong M et al |
|  | rs2274319 | C | T | NA | -0.0139 | 0.0022 | 4.09E-10 | 395781 | NA | 39.92 | Chong M et al |
|  | rs2038480 | T | A | NA | 0.0162 | 0.0027 | 1.39E-09 | 395781 | NA | 36.00 | Chong M et al |
|  | rs10749636 | A | G | NA | 0.0155 | 0.0025 | 5.43E-10 | 395781 | NA | 38.44 | Chong M et al |
|  | rs62641680 | A | G | NA | -0.0903 | 0.0063 | 4.63E-47 | 395781 | NA | 205.44 | Chong M et al |
|  | rs74874677 | G | A | NA | -0.0819 | 0.0071 | 3.62E-31 | 395781 | NA | 133.06 | Chong M et al |
|  | rs12052715 | G | C | NA | -0.0133 | 0.0024 | 1.72E-08 | 395781 | NA | 30.71 | Chong M et al |
|  | rs13084580 | T | C | NA | 0.0244 | 0.0033 | 2.19E-13 | 395781 | NA | 54.67 | Chong M et al |
|  | rs1354034 | C | T | NA | -0.0268 | 0.0022 | 2.05E-35 | 395781 | NA | 148.40 | Chong M et al |
|  | rs13088724 | A | G | NA | 0.0175 | 0.0026 | 7.70E-12 | 395781 | NA | 45.30 | Chong M et al |
|  | rs4698839 | T | C | NA | 0.0124 | 0.0022 | 9.47E-09 | 395781 | NA | 31.77 | Chong M et al |
|  | rs7705526 | A | C | NA | 0.0178 | 0.0023 | 6.46E-15 | 395781 | NA | 59.89 | Chong M et al |
|  | rs114694170 | C | T | NA | 0.0331 | 0.0045 | 3.04E-13 | 395781 | NA | 54.10 | Chong M et al |
|  | rs212930 | G | A | NA | 0.0149 | 0.0025 | 3.18E-09 | 395781 | NA | 35.52 | Chong M et al |
|  | rs5745582 | T | C | NA | 0.0210 | 0.0028 | 3.46E-14 | 395781 | NA | 56.25 | Chong M et al |
|  | rs4895441 | G | A | NA | 0.0177 | 0.0024 | 8.31E-14 | 395781 | NA | 54.39 | Chong M et al |
|  | rs2304693 | A | G | NA | 0.0181 | 0.0028 | 5.11E-11 | 395781 | NA | 41.79 | Chong M et al |
|  | rs11764390 | A | G | NA | -0.0122 | 0.0022 | 1.55E-08 | 395781 | NA | 30.75 | Chong M et al |
|  | rs445 | T | C | NA | 0.0207 | 0.0036 | 8.85E-09 | 395781 | NA | 33.06 | Chong M et al |
|  | rs342293 | G | C | NA | 0.0283 | 0.0021 | 1.05E-40 | 395781 | NA | 181.61 | Chong M et al |
|  | rs6959832 | A | G | NA | -0.0210 | 0.0021 | 2.10E-23 | 395781 | NA | 100.00 | Chong M et al |
|  | rs4284061 | A | T | NA | -0.0186 | 0.0022 | 2.97E-17 | 395781 | NA | 71.48 | Chong M et al |
|  | rs4841132 | G | A | NA | -0.0212 | 0.0037 | 7.46E-09 | 395781 | NA | 32.83 | Chong M et al |
|  | rs2322718 | G | T | NA | 0.0136 | 0.0021 | 1.53E-10 | 395781 | NA | 41.94 | Chong M et al |
|  | rs385893 | C | T | NA | 0.0153 | 0.0021 | 5.96E-13 | 395781 | NA | 53.08 | Chong M et al |
|  | rs8176645 | A | T | NA | -0.0145 | 0.0026 | 3.06E-08 | 395781 | NA | 31.10 | Chong M et al |
|  | rs12247015 | G | A | NA | 0.0337 | 0.0021 | 1.28E-55 | 395781 | NA | 257.53 | Chong M et al |
|  | rs7896518 | G | A | NA | -0.0456 | 0.0022 | 9.53E-99 | 395781 | NA | 429.62 | Chong M et al |
|  | rs57066921 | G | T | NA | -0.1081 | 0.0081 | 8.98E-41 | 395781 | NA | 178.11 | Chong M et al |
|  | rs750866 | G | A | NA | -0.0254 | 0.0026 | 1.27E-22 | 395781 | NA | 95.44 | Chong M et al |
|  | rs11064074 | T | C | NA | 0.0197 | 0.0021 | 4.87E-20 | 395781 | NA | 88.00 | Chong M et al |
|  | rs5012419 | G | A | NA | 0.0247 | 0.0022 | 2.66E-29 | 395781 | NA | 126.05 | Chong M et al |
|  | rs1127787 | A | G | NA | -0.0159 | 0.0028 | 1.49E-08 | 395781 | NA | 32.25 | Chong M et al |
|  | rs2015599 | A | G | NA | 0.0123 | 0.0021 | 6.03E-09 | 395781 | NA | 34.31 | Chong M et al |
|  | rs12426673 | T | G | NA | -0.0141 | 0.0021 | 4.03E-11 | 395781 | NA | 45.08 | Chong M et al |
|  | rs11553699 | G | A | NA | 0.0445 | 0.0032 | 1.45E-43 | 395781 | NA | 193.38 | Chong M et al |
|  | rs1760940 | C | A | NA | 0.0263 | 0.0024 | 5.20E-27 | 395781 | NA | 120.09 | Chong M et al |
|  | rs72698722 | T | C | NA | -0.0192 | 0.0027 | 1.25E-12 | 395781 | NA | 50.57 | Chong M et al |
|  | rs289713 | A | T | NA | -0.0149 | 0.0027 | 4.14E-08 | 395781 | NA | 30.45 | Chong M et al |
|  | rs200309755 | T | C | NA | -0.0180 | 0.0022 | 1.91E-16 | 395781 | NA | 66.94 | Chong M et al |
|  | rs17850455 | G | C | NA | 0.0910 | 0.0104 | 1.81E-18 | 395781 | NA | 76.56 | Chong M et al |
|  | rs77261872 | T | C | NA | 0.0262 | 0.0032 | 2.28E-16 | 395781 | NA | 67.04 | Chong M et al |
|  | rs28665408 | C | A | NA | 0.0157 | 0.0021 | 2.11E-13 | 395781 | NA | 55.89 | Chong M et al |
|  | rs11085147 | T | C | NA | 0.0756 | 0.0036 | 1.54E-95 | 395781 | NA | 441.00 | Chong M et al |
|  | rs6511720 | T | G | NA | 0.0192 | 0.0033 | 4.08E-09 | 395781 | NA | 33.85 | Chong M et al |
|  | rs56069439 | A | C | NA | -0.0274 | 0.0023 | 2.30E-32 | 395781 | NA | 141.92 | Chong M et al |
|  | rs1065853 | T | G | NA | 0.0388 | 0.0039 | 1.59E-23 | 395781 | NA | 98.98 | Chong M et al |
|  | rs1613662 | A | G | NA | -0.0167 | 0.0028 | 3.90E-09 | 395781 | NA | 35.57 | Chong M et al |
|  | rs156355 | C | T | NA | 0.0214 | 0.0022 | 6.41E-23 | 395781 | NA | 94.62 | Chong M et al |
|  | rs4814776 | A | C | NA | -0.0300 | 0.0023 | 2.00E-40 | 395781 | NA | 170.13 | Chong M et al |
|  | rs6105852 | A | G | NA | 0.0221 | 0.0021 | 1.41E-25 | 395781 | NA | 110.75 | Chong M et al |
|  | rs2245946 | A | G | NA | 0.0317 | 0.0023 | 9.38E-45 | 395781 | NA | 189.96 | Chong M et al |
|  | rs12148 | G | T | NA | -0.0139 | 0.0022 | 1.32E-10 | 395781 | NA | 39.92 | Chong M et al |
| **Instrumental variables 2** | |  |  |  |  |  |  |  |  |  |  |
|  | rs4648452 | T | C | 0.1889 | -0.0193 | 0.0033 | 5.96E-09 | 295150 | 1.15E-04 | 33.80 | Hägg S et al |
|  | rs1474868 | T | C | 0.4722 | -0.0203 | 0.0026 | 3.96E-15 | 295150 | 2.05E-04 | 60.40 | Hägg S et al |
|  | rs831522 | C | A | 0.3618 | 0.0227 | 0.0027 | 7.69E-17 | 295150 | 2.39E-04 | 70.49 | Hägg S et al |
|  | rs35734242 | C | T | 0.4414 | -0.0143 | 0.0026 | 4.36E-08 | 295150 | 1.00E-04 | 29.61 | Hägg S et al |
|  | rs12500975 | C | T | 0.4304 | -0.0149 | 0.0026 | 1.02E-08 | 295150 | 1.09E-04 | 32.17 | Hägg S et al |
|  | rs518867 | T | C | 0.3598 | -0.0246 | 0.0027 | 1.99E-20 | 295150 | 2.78E-04 | 82.08 | Hägg S et al |
|  | rs2853672 | A | C | 0.4871 | -0.0178 | 0.0026 | 6.55E-12 | 295150 | 1.58E-04 | 46.52 | Hägg S et al |
|  | rs114694170 | C | T | 0.0537 | 0.0491 | 0.0055 | 3.27E-19 | 295150 | 2.45E-04 | 72.38 | Hägg S et al |
|  | rs2057657 | G | A | 0.2256 | -0.0179 | 0.0030 | 3.81E-09 | 295150 | 1.12E-04 | 33.11 | Hägg S et al |
|  | rs210143 | T | C | 0.2734 | -0.0353 | 0.0028 | 3.13E-36 | 295150 | 4.96E-04 | 146.43 | Hägg S et al |
|  | rs4895441 | G | A | 0.2734 | 0.0315 | 0.0029 | 8.51E-28 | 295150 | 3.94E-04 | 116.39 | Hägg S et al |
|  | rs6976396 | A | C | 0.1759 | -0.0211 | 0.0035 | 2.25E-09 | 295150 | 1.29E-04 | 38.13 | Hägg S et al |
|  | rs12155038 | A | G | 0.4443 | -0.0147 | 0.0026 | 1.59E-08 | 295150 | 1.06E-04 | 31.29 | Hägg S et al |
|  | rs8179 | T | C | 0.2207 | -0.0241 | 0.0032 | 2.89E-14 | 295150 | 2.01E-04 | 59.20 | Hägg S et al |
|  | rs342293 | G | C | 0.4374 | 0.0221 | 0.0026 | 1.13E-17 | 295150 | 2.40E-04 | 70.85 | Hägg S et al |
|  | rs77236693 | T | C | 0.0815 | 0.0303 | 0.0044 | 6.21E-12 | 295150 | 1.37E-04 | 40.58 | Hägg S et al |
|  | rs3110823 | C | A | 0.1481 | 0.0291 | 0.0034 | 1.93E-17 | 295150 | 2.13E-04 | 62.99 | Hägg S et al |
|  | rs4841132 | A | G | 0.0736 | 0.0265 | 0.0045 | 2.87E-09 | 295150 | 9.56E-05 | 28.21 | Hägg S et al |
|  | rs10094039 | A | G | 0.3718 | -0.0175 | 0.0026 | 4.06E-11 | 295150 | 1.43E-04 | 42.15 | Hägg S et al |
|  | rs385893 | T | C | 0.4930 | -0.0215 | 0.0026 | 7.21E-17 | 295150 | 2.31E-04 | 68.25 | Hägg S et al |
|  | rs56225686 | A | T | 0.0507 | -0.0323 | 0.0054 | 1.64E-09 | 295150 | 1.00E-04 | 29.66 | Hägg S et al |
|  | rs11006121 | T | C | 0.3996 | 0.0185 | 0.0026 | 1.58E-12 | 295150 | 1.65E-04 | 48.71 | Hägg S et al |
|  | rs10740118 | C | G | 0.4344 | -0.0270 | 0.0026 | 4.53E-25 | 295150 | 3.58E-04 | 105.80 | Hägg S et al |
|  | rs1408343 | G | A | 0.2336 | -0.0261 | 0.0029 | 2.41E-19 | 295150 | 2.44E-04 | 72.03 | Hägg S et al |
|  | rs10835226 | T | C | 0.3181 | 0.0178 | 0.0029 | 4.90E-10 | 295150 | 1.38E-04 | 40.60 | Hägg S et al |
|  | rs35979828 | T | C | 0.0855 | -0.0341 | 0.0051 | 1.98E-11 | 295150 | 1.82E-04 | 53.60 | Hägg S et al |
|  | rs4388979 | G | T | 0.3767 | 0.0191 | 0.0026 | 3.20E-13 | 295150 | 1.71E-04 | 50.36 | Hägg S et al |
|  | rs3809272 | A | G | 0.3052 | -0.0234 | 0.0028 | 6.61E-17 | 295150 | 2.32E-04 | 68.38 | Hägg S et al |
|  | rs11615667 | A | C | 0.0905 | 0.0268 | 0.0041 | 4.76E-11 | 295150 | 1.18E-04 | 34.85 | Hägg S et al |
|  | rs1760940 | C | A | 0.2455 | 0.0281 | 0.0030 | 3.55E-21 | 295150 | 2.93E-04 | 86.44 | Hägg S et al |
|  | rs59488041 | A | T | 0.1511 | -0.0322 | 0.0038 | 1.18E-17 | 295150 | 2.67E-04 | 78.70 | Hägg S et al |
|  | rs3087374 | A | C | 0.0855 | 0.0270 | 0.0047 | 1.08E-08 | 295150 | 1.14E-04 | 33.55 | Hägg S et al |
|  | rs11865642 | C | A | 0.1829 | -0.0184 | 0.0033 | 3.06E-08 | 295150 | 1.01E-04 | 29.77 | Hägg S et al |
|  | rs12924138 | T | G | 0.3887 | 0.0146 | 0.0026 | 1.24E-08 | 295150 | 1.02E-04 | 30.08 | Hägg S et al |
|  | rs2063185 | T | C | 0.3141 | 0.0166 | 0.0029 | 6.20E-09 | 295150 | 1.18E-04 | 34.92 | Hägg S et al |
|  | rs12451555 | G | T | 0.2445 | -0.0169 | 0.0030 | 1.98E-08 | 295150 | 1.06E-04 | 31.25 | Hägg S et al |
|  | rs11078935 | G | T | 0.3559 | 0.0314 | 0.0027 | 1.10E-31 | 295150 | 4.53E-04 | 133.74 | Hägg S et al |
|  | rs16978036 | T | G | 0.1461 | 0.0262 | 0.0039 | 1.06E-11 | 295150 | 1.71E-04 | 50.56 | Hägg S et al |
|  | rs1790961 | T | G | 0.4761 | -0.0181 | 0.0026 | 2.10E-12 | 295150 | 1.63E-04 | 48.25 | Hägg S et al |
|  | rs806709 | A | G | 0.0855 | 0.0704 | 0.0042 | 3.03E-62 | 295150 | 7.75E-04 | 228.79 | Hägg S et al |
|  | rs10419397 | A | G | 0.2604 | -0.0278 | 0.0028 | 7.40E-23 | 295150 | 2.97E-04 | 87.60 | Hägg S et al |
|  | rs1065853 | T | G | 0.0626 | 0.0376 | 0.0047 | 1.64E-15 | 295150 | 1.66E-04 | 49.11 | Hägg S et al |
|  | rs1613662 | G | A | 0.1451 | 0.0243 | 0.0035 | 1.96E-12 | 295150 | 1.47E-04 | 43.28 | Hägg S et al |
|  | rs11696739 | A | G | 0.3767 | -0.0174 | 0.0026 | 5.57E-11 | 295150 | 1.42E-04 | 41.82 | Hägg S et al |
|  | rs156333 | A | G | 0.4841 | 0.0205 | 0.0026 | 3.64E-15 | 295150 | 2.10E-04 | 61.87 | Hägg S et al |
|  | rs11697739 | T | C | 0.4821 | -0.0254 | 0.0026 | 5.40E-23 | 295150 | 3.23E-04 | 95.22 | Hägg S et al |
|  | rs75107793 | A | G | 0.0587 | 0.0445 | 0.0050 | 4.66E-19 | 295150 | 2.18E-04 | 64.41 | Hägg S et al |
| **Instrumental variables 3** | |  |  |  |  |  |  |  |  |  |  |
|  | rs10411696 | T | G | NA | 0.011692 | 0.002102 | 2.30E-08 | 465809 | NA | 30.94 | Longchamps RJ et al |
|  | rs10419397 | G | A | NA | 0.032272 | 0.002301 | 7.10E-46 | 465809 | NA | 196.71 | Longchamps RJ et al |
|  | rs10749636 | G | A | NA | -0.01532 | 0.002477 | 1.10E-09 | 465809 | NA | 38.25 | Longchamps RJ et al |
|  | rs11085147 | C | T | NA | -0.09148 | 0.003632 | 3.00E-141 | 465809 | NA | 634.40 | Longchamps RJ et al |
|  | rs1127787 | G | A | NA | 0.018808 | 0.002796 | 3.50E-11 | 465809 | NA | 45.25 | Longchamps RJ et al |
|  | rs114694170 | T | C | NA | -0.03657 | 0.004514 | 9.90E-16 | 465809 | NA | 65.63 | Longchamps RJ et al |
|  | rs11553699 | A | G | NA | -0.05075 | 0.003194 | 1.20E-57 | 465809 | NA | 252.47 | Longchamps RJ et al |
|  | rs11668201 | A | T | NA | 0.015043 | 0.00269 | 9.60E-09 | 465809 | NA | 31.27 | Longchamps RJ et al |
|  | rs117728810 | G | A | NA | 0.023917 | 0.004571 | 8.70E-08 | 465809 | NA | 27.38 | Longchamps RJ et al |
|  | rs117948349 | G | A | NA | 0.034522 | 0.005863 | 2.50E-09 | 465809 | NA | 34.67 | Longchamps RJ et al |
|  | rs11867543 | C | T | NA | -0.01846 | 0.003046 | 5.50E-10 | 465809 | NA | 36.73 | Longchamps RJ et al |
|  | rs12052715 | C | G | NA | 0.016754 | 0.002343 | 3.30E-13 | 465809 | NA | 51.13 | Longchamps RJ et al |
|  | rs12148 | T | G | NA | 0.016642 | 0.002151 | 5.70E-15 | 465809 | NA | 59.86 | Longchamps RJ et al |
|  | rs12247015 | A | G | NA | -0.04217 | 0.002126 | 4.40E-89 | 465809 | NA | 393.44 | Longchamps RJ et al |
|  | rs12426673 | G | T | NA | 0.014413 | 0.002127 | 2.20E-11 | 465809 | NA | 45.92 | Longchamps RJ et al |
|  | rs13084580 | C | T | NA | -0.02691 | 0.003309 | 4.20E-16 | 465809 | NA | 66.14 | Longchamps RJ et al |
|  | rs1354034 | T | C | NA | 0.031093 | 0.002136 | 3.40E-49 | 465809 | NA | 211.90 | Longchamps RJ et al |
|  | rs1362214 | A | G | NA | -0.0242 | 0.002113 | 8.50E-31 | 465809 | NA | 131.17 | Longchamps RJ et al |
|  | rs142158911 | G | A | NA | -0.01751 | 0.003301 | 4.60E-08 | 465809 | NA | 28.14 | Longchamps RJ et al |
|  | rs151234 | G | C | NA | 0.020078 | 0.003118 | 7.80E-11 | 465809 | NA | 41.47 | Longchamps RJ et al |
|  | rs156355 | T | C | NA | -0.02446 | 0.002158 | 1.60E-30 | 465809 | NA | 128.47 | Longchamps RJ et al |
|  | rs1569419 | T | C | NA | -0.02295 | 0.002504 | 1.90E-19 | 465809 | NA | 84.00 | Longchamps RJ et al |
|  | rs1613662 | G | A | NA | 0.019946 | 0.002821 | 5.40E-13 | 465809 | NA | 49.99 | Longchamps RJ et al |
|  | rs1716505 | C | G | NA | 0.01328 | 0.002285 | 4.70E-09 | 465809 | NA | 33.78 | Longchamps RJ et al |
|  | rs17260734 | T | A | NA | -0.01124 | 0.002114 | 7.10E-08 | 465809 | NA | 28.27 | Longchamps RJ et al |
|  | rs1760940 | A | C | NA | -0.0271 | 0.00243 | 2.00E-29 | 465809 | NA | 124.37 | Longchamps RJ et al |
|  | rs17850455 | C | G | NA | -0.12051 | 0.010246 | 5.20E-33 | 465809 | NA | 138.34 | Longchamps RJ et al |
|  | rs193541 | C | T | NA | -0.01303 | 0.002122 | 6.10E-10 | 465809 | NA | 37.70 | Longchamps RJ et al |
|  | rs1967556 | T | G | NA | -0.02287 | 0.002107 | 1.30E-27 | 465809 | NA | 117.82 | Longchamps RJ et al |
|  | rs2015599 | G | A | NA | -0.01207 | 0.002104 | 1.50E-08 | 465809 | NA | 32.91 | Longchamps RJ et al |
|  | rs204071 | C | T | NA | -0.02249 | 0.004152 | 2.80E-08 | 465809 | NA | 29.34 | Longchamps RJ et al |
|  | rs2241942 | G | A | NA | 0.013837 | 0.002463 | 1.70E-08 | 465809 | NA | 31.56 | Longchamps RJ et al |
|  | rs2245947 | G | T | NA | -0.03896 | 0.002245 | 2.50E-68 | 465809 | NA | 301.17 | Longchamps RJ et al |
|  | rs2426092 | A | C | NA | -0.01235 | 0.002109 | 3.90E-09 | 465809 | NA | 34.29 | Longchamps RJ et al |
|  | rs261290 | T | C | NA | -0.01257 | 0.002209 | 1.80E-08 | 465809 | NA | 32.38 | Longchamps RJ et al |
|  | rs2736100 | C | A | NA | 0.01726 | 0.002098 | 1.60E-16 | 465809 | NA | 67.68 | Longchamps RJ et al |
|  | rs2844484 | A | G | NA | 0.017398 | 0.002154 | 3.90E-16 | 465809 | NA | 65.24 | Longchamps RJ et al |
|  | rs28665408 | A | C | NA | -0.02023 | 0.00212 | 4.70E-21 | 465809 | NA | 91.06 | Longchamps RJ et al |
|  | rs289713 | T | A | NA | 0.01878 | 0.002696 | 2.50E-12 | 465809 | NA | 48.52 | Longchamps RJ et al |
|  | rs3087374 | C | A | NA | -0.02366 | 0.003836 | 2.60E-10 | 465809 | NA | 38.04 | Longchamps RJ et al |
|  | rs3110823 | A | C | NA | -0.03294 | 0.002793 | 5.90E-33 | 465809 | NA | 139.09 | Longchamps RJ et al |
|  | rs342293 | C | G | NA | -0.03451 | 0.002101 | 7.00E-61 | 465809 | NA | 269.80 | Longchamps RJ et al |
|  | rs34592828 | G | A | NA | -0.03215 | 0.005056 | 5.80E-11 | 465809 | NA | 40.43 | Longchamps RJ et al |
|  | rs34894010 | C | G | NA | -0.03746 | 0.005212 | 1.60E-12 | 465809 | NA | 51.66 | Longchamps RJ et al |
|  | rs3818157 | G | A | NA | 0.021182 | 0.00212 | 3.00E-24 | 465809 | NA | 99.83 | Longchamps RJ et al |
|  | rs385893 | T | C | NA | -0.01432 | 0.002099 | 1.20E-12 | 465809 | NA | 46.54 | Longchamps RJ et al |
|  | rs4284061 | T | A | NA | 0.020073 | 0.002187 | 6.30E-20 | 465809 | NA | 84.24 | Longchamps RJ et al |
|  | rs4427713 | T | C | NA | 0.011989 | 0.002118 | 5.90E-09 | 465809 | NA | 32.04 | Longchamps RJ et al |
|  | rs445 | C | T | NA | -0.02067 | 0.003546 | 3.00E-09 | 465809 | NA | 33.98 | Longchamps RJ et al |
|  | rs4814776 | C | A | NA | 0.036218 | 0.002236 | 5.40E-59 | 465809 | NA | 262.36 | Longchamps RJ et al |
|  | rs4841132 | A | G | NA | 0.027626 | 0.003654 | 6.10E-14 | 465809 | NA | 57.16 | Longchamps RJ et al |
|  | rs4895441 | A | G | NA | -0.01996 | 0.002355 | 9.50E-18 | 465809 | NA | 71.84 | Longchamps RJ et al |
|  | rs4910886 | G | T | NA | -0.02258 | 0.002217 | 6.20E-25 | 465809 | NA | 103.73 | Longchamps RJ et al |
|  | rs55823018 | C | T | NA | 0.013095 | 0.002253 | 5.90E-09 | 465809 | NA | 33.78 | Longchamps RJ et al |
|  | rs56116444 | T | G | NA | 0.025006 | 0.00398 | 3.80E-10 | 465809 | NA | 39.48 | Longchamps RJ et al |
|  | rs5745582 | C | T | NA | -0.02559 | 0.002737 | 1.20E-21 | 465809 | NA | 87.42 | Longchamps RJ et al |
|  | rs59488041 | T | A | NA | 0.0238 | 0.003071 | 2.50E-14 | 465809 | NA | 60.06 | Longchamps RJ et al |
|  | rs602616 | C | G | NA | 0.019298 | 0.003701 | 3.20E-08 | 465809 | NA | 27.19 | Longchamps RJ et al |
|  | rs62641680 | G | A | NA | 0.107204 | 0.006253 | 1.50E-65 | 465809 | NA | 293.93 | Longchamps RJ et al |
|  | rs6425521 | C | A | NA | -0.02174 | 0.002642 | 2.30E-17 | 465809 | NA | 67.71 | Longchamps RJ et al |
|  | rs655029 | G | A | NA | 0.014052 | 0.002335 | 1.60E-09 | 465809 | NA | 36.22 | Longchamps RJ et al |
|  | rs6580981 | G | A | NA | -0.01423 | 0.002115 | 2.40E-11 | 465809 | NA | 45.27 | Longchamps RJ et al |
|  | rs6778131 | T | A | NA | 0.012015 | 0.002176 | 2.30E-08 | 465809 | NA | 30.49 | Longchamps RJ et al |
|  | rs680478 | C | T | NA | 0.016511 | 0.002435 | 7.70E-12 | 465809 | NA | 45.98 | Longchamps RJ et al |
|  | rs6943701 | A | T | NA | -0.01808 | 0.00275 | 2.50E-11 | 465809 | NA | 43.22 | Longchamps RJ et al |
|  | rs6986601 | A | G | NA | 0.011906 | 0.002103 | 7.70E-09 | 465809 | NA | 32.05 | Longchamps RJ et al |
|  | rs7080536 | G | A | NA | 0.032287 | 0.005223 | 6.30E-10 | 465809 | NA | 38.21 | Longchamps RJ et al |
|  | rs711244 | C | T | NA | 0.013768 | 0.002122 | 1.80E-10 | 465809 | NA | 42.10 | Longchamps RJ et al |
|  | rs7213347 | G | C | NA | 0.013164 | 0.002284 | 2.00E-09 | 465809 | NA | 33.22 | Longchamps RJ et al |
|  | rs73349121 | G | C | NA | 0.133878 | 0.008078 | 4.30E-61 | 465809 | NA | 274.67 | Longchamps RJ et al |
|  | rs7412 | C | T | NA | -0.04013 | 0.003861 | 1.30E-24 | 465809 | NA | 108.03 | Longchamps RJ et al |
|  | rs74472890 | T | C | NA | 0.026591 | 0.00484 | 1.20E-08 | 465809 | NA | 30.18 | Longchamps RJ et al |
|  | rs74750282 | T | C | NA | -0.04784 | 0.003735 | 7.30E-37 | 465809 | NA | 164.06 | Longchamps RJ et al |
|  | rs74874677 | A | G | NA | 0.099827 | 0.007026 | 6.00E-46 | 465809 | NA | 201.87 | Longchamps RJ et al |
|  | rs754169 | T | A | NA | -0.02735 | 0.0021 | 1.20E-38 | 465809 | NA | 169.62 | Longchamps RJ et al |
|  | rs77261872 | C | T | NA | -0.03152 | 0.003178 | 4.50E-23 | 465809 | NA | 98.37 | Longchamps RJ et al |
|  | rs7800558 | T | C | NA | 0.012245 | 0.002126 | 1.20E-08 | 465809 | NA | 33.17 | Longchamps RJ et al |
|  | rs7896518 | A | G | NA | 0.051452 | 0.00214 | 1.90E-127 | 465809 | NA | 578.07 | Longchamps RJ et al |
|  |  |  |  |  |  |  |  |  |  |  |  |
